# Neuraminidase-1: A novel therapeutic target in multistage tumorigenesis

**DOI:** 10.18632/oncotarget.8396

**Published:** 2016-03-27

**Authors:** Fiona Haxho, Ronald J. Neufeld, Myron R. Szewczuk

**Affiliations:** ^1^ Departments of Biomedical and Molecular Sciences, Kingston, Ontario, Canada; ^2^ Department of Chemical Engineering, Queen's University, Kingston, Ontario, Canada

**Keywords:** cancer, Neu1, oseltamivir phosphate, tumor angiogenesis, metastasis

## Abstract

Several of the growth factors and their receptor tyrosine kinases (RTK) such as epidermal growth factor (EGF), platelet-derived growth factor (PDGF), fibroblast growth factor (FGF), vascular endothelial growth factor (VEGF), nerve growth factor (NGF) and insulin are promising candidate targets for cancer therapy. Indeed, tyrosine kinase inhibitors (TKI) have been developed to target these growth factors and their receptors, and have demonstrated dramatic initial responses in cancer therapy. Yet, most patients ultimately develop TKI drug resistance and relapse. It is essential in the clinical setting that the targeted therapies are to circumvent multistage tumorigenesis, including genetic mutations at the different growth factor receptors, tumor neovascularization, chemoresistance of tumors, immune-mediated tumorigenesis and the development of tissue invasion and metastasis. Here, we identify a novel receptor signaling platform linked to EGF, NGF, insulin and TOLL-like receptor (TLR) activations, all of which are known to play major roles in tumorigenesis. The importance of these findings signify an innovative and promising entirely new targeted therapy for cancer. The role of mammalian neuraminidase-1 (Neu1) in complex with matrix metalloproteinase-9 and G protein-coupled receptor tethered to RTKs and TLRs is identified as a major target in multistage tumorigenesis. Evidence exposing the link connecting growth factor-binding and immune-mediated tumorigenesis to this novel receptor-signaling paradigm will be reviewed in its current relationship to cancer.

## INTRODUCTION

Neuraminidase-1 (Neu1) has recently emerged as a central target in sialidase-mediated regulation of tumorigenesis. Recent evidence indicates that Neu1 plays a much more profound role in human cancers than previously expected. This review will first describe the cell-surface molecular platform that controls Neu1 sialidase activity, and discuss its relevance in cancer cell signaling. Second, we will summarize the current understanding of Neu1 activity associated with cancer development, and outline the key roles of Neu1 during various stages of tumorigenesis, including regulation of growth factor receptor signaling, control of TOLL-like receptor (TLR) signaling and immune-mediated tumorigenesis, regulation of epithelial-mesenchymal transition (EMT), metastasis and acquired chemoresistance, and regulation of tumor vascularization.

The molecular pathogeneses and new therapeutic targets with a focus on pancreatic cancer have been eloquently reviewed by Wong and Lemoine [1, 2]. Here, a large number of genetic alterations affect only a few major signaling cascades and processes involved in pancreatic tumorigenesis. Although some of the important signaling pathways, such as those involving rat sarcoma (Ras), epidermal growth factor (EGF) receptor, vascular endothelial growth factor (VEGF), gastrin hormone and matrix metalloproteinase (MMP) have been targeted with clinical therapeutic intent, these targeted therapies have been discouraging in a clinical setting. For examples, the failures of (a) bevacizumab, a humanized antibody against VEGF, in combination with gemcitabine and erlotinib, (b) sorafenib, a multi-targeted kinase inhibitor that inhibits the VEGF receptor, platelet-derived growth factor receptor (PDGFR), stem cell factor receptor/c-Kit, Raf-1 proto-oncogene, serine/threonine kinase (RAF1) and Fms-like tyrosine kinase-3 (FLT-3), (c) axitinib, an orally active inhibitor of both VEGFR and related tyrosine kinase receptors, and many more anti-cancer agents, collectively demonstrate the difficulty in the specific targeting and killing of cancer cells [2].

The mutational expression of EGF receptor in cancer cells has been identified in a variety of human tumors, including lung, breast, head and neck, ovarian and pancreatic cancers [3, 4]. These altered EGFRs have been reported to promote cell survival, proliferation, invasion, and metastasis through activation of Janus kinase/signal transducers and activators of transcription (JAK/STAT), phosphoinositol 3-kinase (PI3K), serine/threonine-specific protein kinase-B (Akt), and mitogen activated protein kinase (MAPK) signaling pathways [4–6]. The PI3K/Akt signaling pathway is an important intracellular regulator of the cell cycle. PI3K activation phosphorylates and activates Akt, localizing it in the plasma membrane [7]. Activated Akt in turn affects a number of downstream signaling pathways, such as (a) the cellular transcriptional factor cAMP response element-binding protein (CREB), (b) inhibiting the tumor suppressor cyclin-dependent kinase inhibitor-1B (p27), (c) localizing O subclass of the forkhead family of transcription factors (FOXO) in the cytoplasm, (d) phosphorylating phosphoinositides [PtdIns-(4,5) P2] at the 3′ position of the inositol ring to generate PtdIns-[3,4,5] P3 (PtdIns-3P), and (e) activating the mammalian target of rapamycin (mTOR) which is a master regulator of cell growth and division responding to a variety of stimuli, such as nutrient, energy, and growth factors. The PI3K/Akt signaling pathways have been reviewed in detail [7–9]. Several other factors are known to enhance the PI3K/Akt signaling pathway, including EGF [10, 11], sonic hedgehog (shh) pathway [12], insulin growth factor-1 (IGF-1) [13], insulin [14–16], and calcium/calmodulin (CaM)-dependent protein kinases [17]. The PI3K/Akt pathway is controlled by various antagonistic factors such as the tumor suppressor phosphatase and tensin homolog protein (PTEN) [18–20], glycogen synthase kinase-3β (Gsk3β), a negative modulator in endothelial cells through the Wnt/β-catenin/PI3K/AKT/Gsk3β signaling axis in cancer-induced angiogenesis [21, 22], and the promoter of homeobox gene *HB9* [23]. In many cancers, this PI3K/Akt pathway is overactive by allowing proliferation and reducing apoptosis. Logistically, it follows that tyrosine kinase receptors such as EGFR and others are promising candidate targets for cancer therapy and have led to the development of the tyrosine kinase inhibitors, such as the EGFR-targeting gefitinib and erlotinib. Despite the dramatic initial responses to these inhibitors, most patients ultimately develop drug resistance and relapse.

Drug resistance in over 50% of cancers is caused by a mutation in the adenosine triphosphate (ATP) binding pocket of the EGFR kinase domain [24]. This ATP mutation involves an amino acid substitution within the domain, changing a small polar threonine residue with a large nonpolar methionine residue (T790M). Other instances of drug resistance can involve (a) amplification of the hepatocyte growth factor receptor, which drives human epidermal growth factor receptor-3 (HER3 or ERBB3)-dependent activation of PI3K [25, 26], (b) numerous mutations, including recruitment of a mutated IGF-I receptor to dimerize with EGFR in forming a heterodimer [27] and allowing activation of the downstream effectors of EGFR even in the presence of an EGFR inhibitor, and (c) inactivating mutations of the PTEN tumor suppressor, which allow increased activation of Akt-independent stimulation by EGFR [28]. A recent review by Lin et al. [29] summarizes the activating mutations located in the tyrosine kinase domains of EGFR and the major mechanisms of EGFR acquired resistance against tyrosine kinase inhibitors (TKIs). For a clinically significant anticancer response, treatment strategies must target and inhibit several oncogenic pathways simultaneously, or at multiple levels of a major signaling pathway [1, 2]. For a truly effective clinical outcome, it is essential that these targeted therapies are to circumvent the genetic mutations at different growth factor receptor levels, tumor neovascularization, chemoresistance of tumors, and the development of metastases.

## A NOVEL EGFR-SIGNALING PLATFORM AND ITS TARGETED TRANSLATION IN CANCER

**Figure 1:**
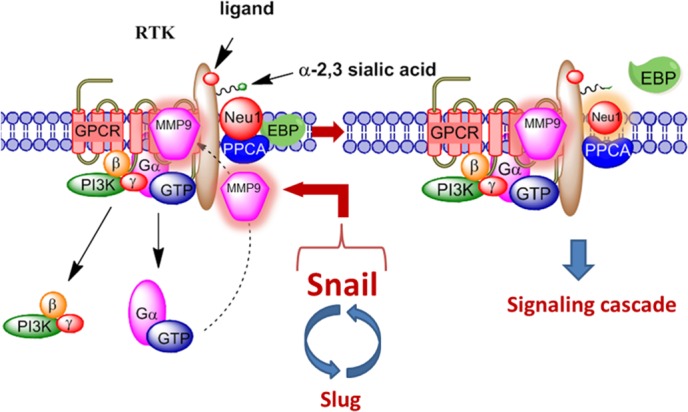
Neuraminidase-1 (Neu1) and matrix metalloproteinase-9 (MMP9) cross-talk in alliance with G protein-coupled receptor(s) (GPCR) regulates receptor tyrosine kinases (RTKs) Notes: Snail and MMP9 expressions are closely connected in invasive tumor processes. Snail induces MMP9 secretion via multiple signaling pathways, but particularly in cooperation with oncogenic H-Ras (RasV12), Snail up-regulates the transcription of MMP9. This Snail-MMP9 signaling axis is the connecting link to promote RTK glycosylation modification involving this novel receptor-signaling platform. Activated MMP9 is proposed to remove the elastin-binding protein (EBP) as part of the molecular multi-enzymatic complex that contains β-galactosidase/Neu1 and protective protein cathepsin A (PPCA) to induce Neu1. Activated Neu1 hydrolyzes α-2,3-sialic acid residues of the glycosylated receptors at the ectodomain to remove steric hindrance and to facilitate receptor association and activation. This process sets the stage for multistages of tumorigenesis. Abbreviations: Neu1, neuraminidase-1; MMP, matrix metalloproteinase; PI3K, phosphatidylinositol 3-kinase; GTP, guanine triphosphate; GPCR, G protein-coupled receptor; EBP, elastin binding protein; PPCA, protective protein cathepsin A. Citation: Taken in part from: ©Abdulkhalek et al. Research and Reports in Biochemistry 2013:3,17–30, and ©Abdulkhalek et al. Clinical and Translational Medicine 2014:3,28. Publisher and licensee Dove Medical Press Ltd. This is an Open Access article which permits unrestricted non-commercial use, provided the original work is properly cited.

A molecular organizational G protein-coupled receptor (GPCR)-signaling platform was uncovered by us that was deemed essential for the activation of EGFR and its targeted translation in pancreatic cancer [30]. This receptor signaling paradigm is depicted in Figure 1, and is described in detail by Abdulkhalek et al. [31]. Here, EGF binding to its receptor has been shown to induce an endogenous mammalian Neu1 and matrix metalloproteinase-9 (MMP9) cross-talk in activating the receptor. Central to this process is that Neu1 and MMP9 form a complex tethered at the ectodomain of EGFRs on the cell surface. This signaling paradigm proposes that EGF binding to its receptor causes a conformational change of EGFR, which results in the activation of neuromedin B GPCR (NMBR) also tethered to the receptor. Activated NMBR initiates Gα_i_-protein signaling which triggers the activation of MMP9 to subsequently induce Neu1. Here, activated MMP9 is proposed to remove the elastin-binding protein (EBP) as part of the molecular multi-enzymatic complex that contains Neu1 and protective protein cathepsin A (PPCA) [32]. Activated Neu1 specifically hydrolyzes the α-2,3-sialyl residues linked to β-galactosides of EGFR, which are distant from the EGF binding sites. This prerequisite desialylation process by Neu1 is predicted to remove steric hindrance of EGFR to facilitate receptor association, subsequent activation and downstream signaling.

At the genetic level, we reported that the sialidase activity associated with EGF stimulation of human 1140F01 and WG0544 type 1 sialidosis fibroblast cell lines was completely abrogated compared to the wild-type fibroblast cell line [30]. These sialidosis fibroblast cells were obtained from patients with type 1 sialidosis or mucolipidosis-1 who have a true Neu1 deficiency [33].

**Figure 2:**
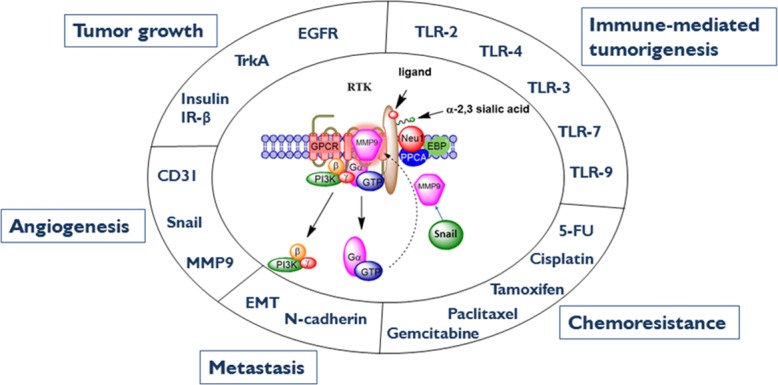
Neu1-MMP9-GPCR signaling platform in the regulation of RTK and the molecular targeting of multistage tumorigenesis Neuraminidase-1 (Neu1) and matrix metalloproteinase-9 (MMP9) cross-talk in alliance with G protein-coupled receptor(s) (GPCR) regulates receptor tyrosine kinases (RTKs) and extracellular and intracellular TOLL-like (TLR) receptors in cancer cells. This process sets the stage for multistage tumorigenesis. Abbreviations: Neu1, neuraminidase-1; MMP, matrix metalloproteinase; IRβ, insulin receptor β; EGCR, epidermal growth factor receptor; EMT, epithelial-mesenchymal transition; 5-FU, 5-fluorouracil; PI3K, phosphatidylinositol 3-kinase; GTP, guanine triphosphate; GPCR, G protein-coupled receptor; EBP, elastin binding protein; PPCA, protective protein cathepsin A. Citation: Taken in part from: ©Abdulkhalek et al. Research and Reports in Biochemistry 2013:3,17–30, and ©Abdulkhalek et al. Clinical and Translational Medicine 2014:3,28. Publisher and licensee Dove Medical Press Ltd. This is an Open Access article which permits unrestricted non-commercial use, provided the original work is properly cited.

In addition, oseltamivir phosphate was found to target and inhibit Neu1 activity associated with the activation of glycosylated receptors by their ligands [34, 35]. However, it is noted that oseltamivir phosphate may also have broader specificity for other sialidases, and thus, the therapeutic effects of oseltamivir phosphate could be due to a multitude of different molecular pathways. For example, in invasive tumors like ovarian cancers, the transcriptional factor Snail and MMP9 expressions are closely connected since they have both been implicated in similar invasive processes [36]. It has been shown that Snail induces MMP9 secretion via multiple signaling pathways, but particularly in cooperation with oncogenic H-Ras (RasV12), Snail leads to the transcriptional upregulation of MMP9 [37]. There is substantial evidence to indicate that the zinc-finger transcriptional factors Snail and Slug, the two-handed zinc factors ZEB1/dEF1 and ZEB2/SIP1, and the basic helix-loop-helix transcription factors Twist and E12/E47 play major roles in epithelial carcinoma plasticity [38–41], and tumor progression and invasiveness [42–45]. Since Snail is identified as a potent EMT mediator, others have reported that it controls the proteolytic activity of the MMPs that contribute to the phenotypic changes associated with EMT and invasion [42]. Taken all together, these different signaling paradigms involved with EMT in ovarian cancer suggest that growth factor receptor glycosylation modification involving the receptor-signaling platform of a Neu1-MMP9 crosstalk may in fact be the invisible link connecting the Snail-MMP9 signaling axis as depicted in Figure 1. It follows that the therapeutic efficacy of oseltamivir phosphate targeting Neu1 may disrupt these molecular signaling pathways. Given the ability of oseltamivir phosphate to increase E-cadherin expression and decrease N-cadherin and VE-cadherin expression as previously reported by us [46], tumors treated with this drug may become more adherent to the surrounding tissue and not metastasize as our data indicated. We propose here a graphical abstract (Figure 2) illustrating that the Snail-MMP9 signaling axis maintains several important cancer growth factor receptor signaling platforms in promoting Neu1-MMP9 crosstalk in complex with glycosylated receptors. Oseltamivir phosphate treatment strategies under dose dependence would take the form of a horizontal approach, of which different oncogenic signaling pathways involved in tumorigenesis are targeted with promising therapeutic intent.

In contrast, other purified neuraminidase inhibitors may not be as potent. For examples, BCX-1827 except BCX-1812, DANA (2-deoxy-2,3-dehydro-N-acetyl-neuraminic acid), zanamivir (4-guanidino-Neu5Ac2en), and oseltamivir carboxylate had limited significant inhibition of lipopolysaccharide (LPS)-induced sialidase activity in live BMC-2 macrophage cells at 1–2 mM compared to the LPS positive control [34]. Oseltamivir phosphate had an IC50 value of 4.86 μM for EGFR [30], which is comparable to the reported IC50 values of 3.876 μM for NGF-TrkA [19] and 1.175 μM for LPS-TLR4 [22] ligand-induced sialidase activity in TrkA-PC12 and BMC-2 macrophage cells, respectively. For NGF-induced sialidase activity in TrkA-expressing cells, we also reported that other purified neuraminidase inhibitors such as zanamivir (4-guanidino-Neu5Ac2en) and oseltamivir carboxylate had a limited inhibition of NGF-induced sialidase activity in live TrkA-PC12 cells at 1–2 mM compared to the NGF positive control [35]. Using recombinant soluble human sialidases, Hata et al. [47] have reported that oseltamivir (actually used oseltamivir carboxylate) scarcely inhibited the activities of the four human sialidases even at 1 mM, while zanamivir significantly inhibited the human Neu2 and Neu3 sialidases in the micromolar range. Using lysates from mature dendritic cells, Nan et al. [48] have found that zanamivir completely inhibited Neu1 and Neu3 sialidase activity at 2 mM.

Other reports have provided supporting evidence for a role of Neu1 in the receptor glycosylation modification model in respiratory airway epithelia. Lillehoj et al. [49] have demonstrated that Neu1 associates with EGFR as well as with the cell surface associated mucin-1 (MUC1) in respiratory airway epithelial cells (EC). This Neu1-EGFR association was regulated by EGF stimulation of the cells, which is consistent and fits well with our receptor signaling platform in Figure 1. However, they also found that overexpression of Neu1 using recombinant adenovirus (Ad) encoding FLAG-tagged human NEU1 (Ad-NEU1) diminished EGF-stimulated EGFR Tyr-1068 autophosphorylation by up to 44% but instead, enhanced MUC1-dependent *Pseudomonas aeruginosa* adhesion by about 2-fold and flagellin-stimulated ERK1/2 activation by nearly 2-fold. In contrast, Neu1 depletion by siRNA knockdown increased EGFR activation (1.5-fold) and diminished MUC1-mediated bacterial adhesion (38-56%) and signaling (73%). These latter results are inconsistent with the EGFR signaling platform as depicted in Figure 1. It is noteworthy from their supplementary data that the total EGFR was profoundly diminished following EGF stimulation, irrespective of Neu1 manipulation, which was likely due to ligand-dependent endocytosis and degradation of EGF-EGFR complexes as previously reported by others [50]. Here, the phospho-Tyr-1068 EGFR signal was normalized to β-tubulin expression. However, Lillehoj et al. [49] have proposed several other possibilities and questioned whether Neu1 targets sialic acid residues within the ligand-binding portion of the EGFR ectodomain to influence the receptor-ligand interaction, or it regulates EGFR homo- or hetero-dimerization, and alters EGFR responsiveness to inhibitory gangliosides. The effect of overexpression of Neu1 was suggested to desialylate the terminally sialylated N-linked oligosaccharides to which ganglioside GM3 binds at the ectodomain of EGFR, and thereby promoting the GM3–EGFR interaction and attenuation of EGFR activation [49]. The inhibitory modulation of EGF receptor activity by changes in the GM3 content in epidermoid cell lines has been well documented [51].

The dimerization process of receptors following EGF binding is an essential required step in the receptor activation process, but the mechanism of which was unknown [52–58] until now as depicted in Figure 1. It is noteworthy that we have reported a striking similarity with this novel receptor signaling platform for nerve growth factor (NGF) TrkA receptors [35], insulin [59, 60] and cell surface TOLL-like receptor (TLR)-4, [34, 61–64] and intracellular TLR7 and TLR9 receptors [65], all of which require receptor dimerization and are regulated by Neu1. Pshezhetsky and Ashmarina [66] have recently summarized the emerging data demonstrating that Neu1, well known for its lysosomal catabolic function, is also localized to the cell surface and assumes the previously unrecognized role as a structural and functional modulator of cellular receptors.

Although Lillehoj et al. [49] have provided evidence to show Neu1 associates with EGFR, the effects of NEU1 overexpression in respiratory airway epithelial cells are inconsistent with this new EGFR signaling platform (Figure 1). To explain this inconsistency, there have been attempts in past years to enhance the efficiency of a biological response by overexpressing single enzymatic activities in mammalian cells. These approaches have been successful in some cases by improving cellular protection from endogenous and exogenous agents, while overexpression of other enzymatic activities were detrimental by producing a genome instability phenotype [67, 68]. Perhaps, overexpression of Neu1 in metabolically active cancer cells may produce a variant with different phenotypes. The approach by Gilmour et al. [30] was to investigate Neu1 regulation of EGF-induced receptor phosphorylation using NIH3T3 mouse embryo fibroblast cell line overly expressing the human EGFR (3T3-hEGFR). The data in the report provided strong evidence to support Neu1 regulation of EGF-induced receptor phosphorylation and subsequent activation. Firstly, the neuraminidase inhibitor oseltamivir phosphate as well as anti-Neu1 but not anti-Neu-2, −3 or −4 neutralizing antibodies, inhibited EGF-induced phosphorylation of EGFR (pEGFR) in 3T3–hEGFR cells. Secondly, the treatment protocol had no effect on reducing the expression of EGFR on the cell surface post-EGF stimulation or treatments, suggesting that oseltamivir phosphate, anti-Neu1 antibodies as well as the specific MMP9 inhibitor had a direct inhibitory effect on the Neu1 activity associated with EGF treated cells, and it was not due to an internalization of EGF-stimulated receptors. Thirdly, *M. amurensis* lectin MAL-2 (specific for α-2,3 sialic acid linked to terminal β-galactose) significantly blocked EGF-induced pEGFR dose-dependently, but had no effect on Neu1 activation. In contrast, *S. nigra* lectin (SNA, which binds to α-2,6 sialic acid linked to terminal galactose and to lesser degree α-2,3 linkage), peanut agglutinin (PNA, galactosyl (β-1,3) N-acetylgalactosamine) and succinylated wheat germ agglutinin (sWGA, N-acetylglucosamine residues) had little effect on blocking EGF-induced pEGFR activation. Fourthly, co-immunoprecipitation experiments using cell lysates from 3T3–hEGFR cells demonstrated that MMP9 forms a complex with naïve and EGF-stimulated EGFRs, and western blot analyses clearly showed that MMP9i inhibited EGF-induced pEGFR in these cell lysates. Fifthly, the anti-cancer role of oseltamivir phosphate was also investigated in human pancreatic tumor-bearing RAGxCγ double mutant mice. Using western blot analyses for pEGFR, pStat1, and pNFκB in the tumor lysates on individual tumors taken from the untreated and oseltamivir phosphate treated cohorts, the data indicated a remarkable significant inhibition of pEGFR and downstream pNFκB and pStat1 activity in the tumor lysates from oseltamivir phosphate treated tumor-bearing mice compared to the untreated cohort. To validate the Western blot analyses, Bio-Plex phospho-protein multiplex analyses showed that simultaneously examined phospho-protein end-points of Akt-Thr308, PDGFRα-Tyr754 and STAT1-Tyr701 were diminished in the tumor lysates from oseltamivir phosphate treated cohort compared to the untreated group. In contrast, oseltamivir phosphate treatment increased the phospho-protein end-points of SMAD2-Ser465/467 and VEGFR2-Tyr1175 compared to the untreated cohort. Collectively, the additional intracellular and cell surface colocalization of Neu1 and MMP9 validated the predicted cross-talk between the neuromedin B GPCR–MMP9–Neu1 tripartite tethered to EGF receptors.

Since the activity of Neu1 tethered to EGFR hydrolyzes α-2,3-sialyl residues exposing terminal β-galactosides, Gilmour et al. [30] also questioned whether mammalian lectins would be recruited to stabilize pEGFR. Confocal microscopy validated the predicted association of galectin-3 with EGF receptors in naïve (26% overlay) and EGF-treated (86% overlay) 3T3–hEGFR cells. To confirm these results, co-immunoprecipitation experiments using cell lysates from 3T3–EGFR cells further validated that galectin-3 forms a complex with EGF-stimulated receptors as predicted. In support of this hypothesis, Zhao et al. [69] have also shown that activated EGFRs are anchored on the cell surface by a galectin-3 lattice, leading to the positive regulation of EGFR signals. Other reports have demonstrated that galectin-3 is a member of a large family of β-galactoside-binding lectins on the cell surface glycoproteins [70], and its expression necessitates tyrosine kinase phosphorylation [71]. Galectin-3 is characteristically localized in the cytosol but possesses the ability to cross intracellular and plasma membranes to translocate into the nucleus, mitochondria, cell surface or extracellular milieu [72, 73]. We have reported that galectin-3 stabilizes Neu1–MMP9 crosstalk in alliance with neuromedin B GPCR tethered to EGFR at the ectodomain on the cell surface, which is required for EGF-induced activation of EGFR [30]. Indeed, Moody et al. [74] have reported that the neuromedin B GPCR regulates EGFRs by a mechanism dependent on MMP activation, which fits well with our receptor signaling model (Figure 1). It is well known that agonist-induced GPCRs have been shown to activate numerous MMPs [75], including MMP-3 [76], MMP-2 and MMP9 [77, 78], including members of the ADAM family of metalloproteinases [79, 80]. We have shown that GPCR agonists can directly activate Neu1 through the intermediate activity of MMP9 in order to induce transactivation of TLRs and subsequent cellular signaling [62, 65]. These findings are consistent with our GPCR-Neu1-MMP9 signaling axis tethered to glycosylated receptors such as EGFR, Trk, insulin, cell surface and intracellular TLRs.

## ABERRANT SIALYLATION IN CANCER PROGRESSION AND METASTASIS

Over 3-5 decades, altered sialylation of tumor cell surface glycoproteins has been described to be highly associated with the metastatic phenotype of cancer [81–84]. In an effort to understand this metastatic behaviour in relation to altered sialic acid, tumor cell surfaces have been extensively analyzed in the past and present for melanomas [85–93], T-cell hybridomas [94], methylcholanthrene A-induced T-cell lymphoma sublines [95, 96], B16F10 melanoma cells [97], metastatic variants [98] and breast cancer [99]. It is now accepted that aberrant sialylation in cancer cells is at least one of the characteristic features associated with the metastatic potential of cancer cells [93–95, 100].

It is noteworthy from an early study that the spontaneous high-metastatic variant (ESb) of the mouse lymphoma L5178Y, which show high propensity for liver metastases, interacted *in vitro* with the isolated autologous hepatocytes [95, 96]. In contrast, the low-metastatic cells of the same tumor (Eb) did not. The hepatocytes were found to bind the metastatic variant (ESb) cells through a lectin-like hepatic binding protein with molecular weights of 52, 56 and 110 kD, and specificity for D-galactosyl and N-acetyl-D-galactosaminyl residues. The low-metastatic cells (Eb) formed hepatocyte interactions only after neuraminidase pretreatment, indicating that lectin binding carbohydrate structures existed in a cryptic form masked on these cells by sialic acid. These results signify that the metastatic potential of cancer cells may require special sialoglycan structures expressed on the cell surface proteins and lipids. In support of this premise, Passaniti and Hart [101] probed the cell surfaces of several metastatic variants of the murine B16 melanoma that were selected for experimental lung-colonizing ability or for their ability to spontaneously metastasize from the site of a subcutaneous injection. Probing the cell surface saccharide topography for specific oligosaccharides, they found no significant differences between the efficient lung-colonizing variant, B16-F10 and the poorly-colonizing B16-Fl or B16-Flr variants. In contrast, the spontaneously metastatic variants contained substantially different levels of specific sialylation sites. The tumorigenic and non-metastatic B16-LM3/G3.26 variant contained 4-fold more GalP1-3GalNAc sialylation sites than the tumorigenic and highly metastatic B16-LM3/ G3.12 variant. Collectively, these results suggest that the relative levels of specific sialoglycan structures correlated well with the ability of the cells to undergo spontaneous metastasis from a subcutaneous tumor.

Other studies have shown that an aberrant sialylation in metastatic cancer cells may not be the main characteristic feature. Based on gene transfection studies, Sawada et al. [102] proposed that alteration of sialidase expression is not a result of metastasis but rather a determining event affecting the metastatic ability. The sialic acid expression seems to vary from cell to cell. For an example, Miyagi et al. [103] showed results that 3Y1 malignant fibroblasts compared to their parental B16 melanoma cells did not show any significant differences in total cellular and surface sialic acid contents, whereas Sawada et al. [102] showed results with metastatic clones of murine colon adenocarcinoma 26, NL17and NL22, highly metastatic and NL44 and NL4 lowly metastatic that exhibited a decrease in highly metastatic cells as compared with their poorly metastatic counterparts. It is likely that metastasis may not be correlated with the overall sialic acid content, but only with levels of specific molecules that could be targets for endogenous sialidases and/or sialyltransferases. Yogeeswaran and Tao [82] have also shown that the sialic acid content in the low-metastasizing WGAR variant clones of B-16 melanoma cells was reduced compared to the parental ConR and RCAR cells. However, the report also emphasized that the RCAR cells showed decreased metastasizing capacity without significant alteration in the content of surface sialic acid. Perhaps, there are certain sialylated Asn-linked oligosaccharides found on metastatic tumor cells that are required for expression of the metastatic phenotype as proposed by Dennis and Laferte [93]. These structures may be directly associated with β1-6 branching of Asn-linked oligosaccharides [91]. A detailed review by Park and Lee [104] summarizes how β-galactoside α2,6 sialyltransferase (ST6 Gal I) with subsequent elevated levels of cell-surface α2,6 -linked sialic acids have been implicated in the altered expression of sialylated glycoproteins with their linkage to colorectal cancer metastasis, radio-resistance, and chemoresistance.

The expression levels of α2,3-sialic acid residues of 50 primary tumor cases, 50 pair-matched lymph node metastasis tumor samples as well as the MDA-MB-231, T-47D and MCF-7 breast cancer cell lines with different metastatic phenotypes were examined by Cui et al. [99]. Using histochemistry, cytochemistry, flow cytometry with *Maackia amurensis* lectin (MAL, specificity for α2,3-sialic acid), cell adhesion and trans-well *in vitro* assays, the data showed that the pair-matched primary lymph node metastatic tumor samples exhibited significantly higher levels of expression of α2,3-sialic acid residues compared to that of primary tumors. In addition, 81.58% of the primary tumors in T1/T2 stages had weak staining for MAL, whereas of 12 tumor cases in T3/T4 stages, only 1 (8.33%) had weak reactions for MAL. The highly metastatic breast cancer cell line MDA-MB-231 exhibited the strongest binding to MAL and the highest expression levels of α2,3-sialic acid residues among the selected cell lines, and this feature was dependent on the mRNA expression levels of α2,3-sialyltransferase gene. The adhesion, invasion and migration activities confirmed that MDA-MB-231 exhibited the greater cell adhesion to, migration toward and invasion to Matrigel.

Recent reviews by Bull et al. [105, 106] have described the specific tumor characteristics associated with the increased expression of sialic acid sugars on the surface of cancer cells. From a sialic acid perspective, the reviews describe evidence to support the role of sialic acids in cancer. Here, tumor-derived sialic acids have been shown to disable cytotoxicity mechanisms of effector immune cells, trigger production of immune suppressive cytokines and dampen activation of antigen-presenting cells [106]. This aberrant sialylation would indeed favor tumor growth and progression.

To uncover the differences of protein glycosylation and further link them to protein functions, Liu et al. [107] labeled glycosylated proteins with alkyne-sugar probes, followed by copper [I]-catalyzed alkyne-azide click chemistry to identify the sialylated and fucosylated proteins in lung cancer cell lines CL1-0 and CL1-5, both of which are derived from the same parental cell line and having distinct invasion capabilities. The data showed that the more invasive cell line CL1-5 exhibited higher sialylation and fucosylation levels and expressed more sialylated proteins. EGFR in CL1-5 exhibited higher sialylation and fucosylation levels and resulted in lower dimerization and tyrosine phosphorylation than in CL1-0 during EGF stimulation. In addition, removal of sialic acids from EGFR by sialidase increased dimer formation of EGFR upon EGF treatment on the cell, and pretreating EGFR with fucosidase also resulted in a similar dimerization enhancement *in vitro*. Moreover, Yen et al. [108] investigated the effect of sialylation on the phosphorylation profile of EGFR in tyrosine kinase inhibitor (TKI)-sensitive and TKI-resistant cells. They showed that sialylation inhibited the association of EGFR with EGF and the subsequent autophosphorylation. In the absence of EGF, the TKI-resistant EGFR mutant at L858R/T790M had a higher degree of sialylation and phosphorylation at Y1068, Y1086, and Y1173 than the TKI-sensitive EGFR. Although sialylation in the TKI-resistant mutants suppresses EGFR tyrosine phosphorylation with the most significant effect on the Y1173 site, the sialylation effect was not strong enough to stop cancer progression by inhibiting the phosphorylation of these three sites. Collectively, these studies reveal the complexity of EGFR sialylation and phosphorylation process. Although sialylation is induced to suppress the phosphorylation of EGFR, the effect of suppression was not strong enough to inhibit the downstream signaling necessary for cancer progression.

Interference with sialic acid expression in cancer cells has been the current target in preventing cancer metastasis. In particular, desialylation of cancer cells by overexpressing human sialidases has been reported to inhibit metastases in murine metastasis models [109–111]. As discussed above, care must be taken in the interpretation of the data taken from these approaches in that they may produce genomic instability phenotypes [68]. However, these overexpressing techniques have been successful in improving cellular protection from endogenous and exogenous mutagens. In particular, Uemura et al. [109] demonstrated that when the human sialidase gene NEU1 was overexpressed in colon cancer HT-29 cells, and injected trans-splenically into mice, the liver metastasis of the NEU1-overexpressing cells was significantly reduced. *In vitro* studies also showed that overexpressing NEU1 suppressed cell migration, invasion and adhesion, whereas the silencing resulted in the opposite. Further analyses of the desialylation process of colon HT-29 cells suggested that NEU1 may be an important regulator of β4-integrin mediated cellular signaling, leading to suppression of metastasis. Although these findings are inconsistent with other studies [30, 82, 99, 102, 103, 112], there might be other explanations. Since the HT29 cells display an undifferentiated phenotype and were injected directly into the spleen, they may have bypassed the extravasation phase of the metastatic process, and thus only a portion of the total metastatic process is represented by these cells in the spleen as suggested in the past by others [101]. Furthermore, several factors such as oncogenes, hormones, and other compensatory mechanisms may increase the expression of sialyltransferases and downregulate the expression of Neu sialidases in the cancer cells [105]. As a result, sialoglycan synthesis in the Golgi system by sialyltransferases is enhanced, and the hydrolysis of sialoglycans by sialidases in the lysosome is reduced, leading to accumulation of hypersialylated structures on the cell membrane.

Sialyltransferases constitute an attractive target as they have been shown to underlie aberrant cancer sialylation [113, 114]. For an example, Ferreira et al. [114] questioned if the expression of sialyltransferases is different in premalignant and in malignant skin tumors. Their results showed that the high expression of ST3Gal-I (β-galactoside α2,3-sialyltransferase-I) and ST6Gal-I (β-galactoside α2,6 sialyltransferase-I) in skin tumors is associated with tumors with a greater potential for invasion and metastasis, as in the case of squamous cell carcinoma [115]. This phenotype may be related to their metastatic behavior. In addition, Lopez-Morales et al. [116] showed that α2,3-linked sialic acid and α2,6-linked sialic acid increased in intensity and distribution in concordance with low and high squamous intraepithelial neoplasia lesions and in normal tissue. Interestingly, they proposed from their data that the change in sialylation occurs before cancer development and may play an important role in cellular cervix transformation into cancerous cells.

Chang et al. [97] investigated the effects of soyasaponin I (Ssa-I), an inhibitor of sialyltransferases, on tumor metastasis using a highly metastatic cancer cell line B16F10. Ssa-I specifically inhibits the expression of α2,3-linked sialic acids without affecting the other glycans on the B16F10 cell surface. They found that Ssa-I decreased the migratory ability of cells and concomitantly enhanced cell adhesion to extracellular matrix proteins. Furthermore, Park and Lee [104] found that increasing STGal-I (β-galactoside α2,6 sialyltransferase) elevated the levels of cell-surface α2,6-linked sialic acids on proteins which have been associated with metastatic spread and therapeutic resistance in colorectal cancer. Collectively, sialyltransferases are mainly expressed in the Golgi apparatus where they incorporate sialic acid residues into assembling glycan structures of cell surface glycoproteins and lipids. There are more than 20 different human and murine sialyltransferases which have been identified. Hence, the upregulation of sialyltransferases results in the expression of highly sialylated structures including sialoglycoproteins, sialogangliosides, or sialyl Lewis a or x (SLe a/x) antigens.

In addition to sialyltransferases, there are four types of sialidases in mammalian cells, which have been found to behave in different ways during carcinogenesis, and to demonstrate aberrant expression in cancer progression [117–119]. Endogenous mammalian sialidases (alternatively referred to as neuraminidases), are glycohydrolytic enzymes that catalyze the removal of sialic acid residues from glycoproteins and glycolipids [118–121]. To date, there are four types of human sialidases that have been characterized, and are classified according to their subcellular localization: (a) lysosomal and cell membrane (Neu1), (b) cytosolic (Neu2), (c) plasma membrane-bound (Neu3), and (d) lysosomal or mitochondrial-associated (Neu4). While they share structural similarities, they differ in their functions and substrate specificities [121, 122].

Neu1 in the lysosomes is associated with lysosomal carboxypeptidase A (protective protein cathepsin A), β-galactosidase, and N-acetyl-galactosamine-6-sulphate sulphatase [123]. Neu1 functions mainly to regulate lipid storage in lysosomes, but also negatively regulates lysosomal exocytosis in hematopoietic cells where it processes the sialic acids on the lysosomal membrane protein (LAMP-1) [124]. Traditionally, Neu1 has always been classed as a lysosomal enzyme until its presence was discovered at the surface of cells. Elastin binding protein that is involved in elastin fibre deposition, was found to form a complex with Neu1/cathepsin A at the cell surface [125]. In activated lymphocytes, lysosomal sialidase is redistributed to the cell membrane [126]. Here, a nine-fold increase in Neu1-specific activity is detected at the cell surface, where the Neu1-cathepsin A complex influences signaling that results in the production of interferon-γ (IFNγ) [48]. Neu1 expression is also upregulated during monocyte differentiation, and is trafficked to the membrane via MHC class II vesicles [123]. More recently, interactions between sialic acid-binding immunoglobulin-type lectins (Siglecs) and TLRs mediated by Neu1 have been reported [127]. The data indicate that TLR4 activation by endotoxin triggers Neu1 translocation to the cell surface to disrupt TLR4-Siglec-E interaction. Neu1-deficient mice produce markedly less IgE and IgG1 antibodies following immunization with protein antigens, the failure of which is to produce IL-4 cytokines [128].

Cytosolic Neu2 is notably expressed in extremely low or undetectable levels in many human tissues and cells, with notable exceptions like the placenta and testis [129], and at higher levels in skeletal muscle [130], the liver [131], and the thymus [132]. Neu2 has also been shown to play a significant role in myoblast differentiation [133]. The crystal structure of human Neu2 in its free form as well as in complex with the neuraminidase inhibitor 2-deoxy-2,3-dehydro-N-acetylneuraminic acid (DANA) has been characterized [134].

Plasma membrane-bound Neu3 is involved in ganglioside degradation and preferentially targets GM3 gangliosides [135, 136]. Neu3 modification of ganglioside pattern has been implicated in cell-to-cell interactions [137], modulation of GM3 levels in skeletal myoblasts favoring their differentiation and protection from apoptosis [135], and hypoxia activation of Neu3 protecting skeletal muscle cells from apoptosis through the activation of the EGFR signaling and the hypoxia-inducible factor (HIF)-1α [138]. Hepatic Neu3 overexpression was reported to improve insulin sensitivity and glucose tolerance through modification of ganglioside composition and peroxisome proliferator-activated receptor-γ signaling [139]. In mice, the over-expression of Neu3 was implicated in the development of severe insulin-resistant diabetes, and may be an important regulator of insulin sensitivity and glucose tolerance. Membrane Neu3 is also highly expressed in human melanoma cells where it promotes cell growth with minimal changes in the composition of gangliosides [140]. Moreover, other studies have reported a relationship between *NEU3* and *GD3* synthase genes that were significantly up-regulated in melanomas in comparison to melanocytes, possibly as a direct consequence of the increased expression of the transcriptional factor Sp1 [141]. Neu3 could be involved in melanoma malignancy by decreasing the levels of Neu5Ac-GM3. Indeed, Yamaguchi et al. have reported evidence to support plasma-membrane-associated *NEU3* gene regulated by Sp1/Sp3 transcription factors [142].

Neu4 is highly expressed in the mucosal surfaces of the colon, although this expression was markedly reduced in colon cancer, suggesting a protective role for Neu4 in the maintenance of normal colon mucosa [143]. Additionally, Neu4 can also be localized on the cell surface of macrophages [144]. We have reported an unprecedented activation of Neu4 on the cell surface of macrophages, dendritic cells, and normal and type I sialidosis human fibroblast cells by thymoquinone (TQ) [145]. TQ which is derived from the nutraceutical black cumin oil had no inhibitory effect on endotoxin lipopolysaccharide (LPS)-induced sialidase activity in live BMC-2 macrophage cells [145]. In contrast, the parent black seed cumin oil and another constituent para-cymene of the oil completely blocked LPS-induced sialidase activity. All of these compounds had no effect on cell viability. Furthermore, the mechanism of TQ-induced Neu4 activation on the cell surface was determined to involve the potentiation of GPCR-signaling by TQ via membrane targeting of Gα_i_ subunit proteins and MMP9 activation [144].

With a particular focus on cancer, the four types of mammalian sialidases have been described to behave in different manners during carcinogenesis, but their alterations however, may influence or facilitate a malignant phenotype including uncontrolled growth, invasion and metastasis [146]. The report proposed that these sialidases are important enzymes that may redefine cancer malignancy and thus may be potential targets for cancer diagnosis and therapy.

## GPCR SIGNAL INTEGRATION IN RECEPTOR TYROSINE KINASE ACTIVATION

The GPCR signal integration in receptor tyrosine kinase activation has been extensively reviewed by Patel [147] and Abdulkhalek et al. [31]. MMP9 and neuromedin B GPCR (NMBR) are associated with each other on the cell surface, and together form a complex with EGFR [30], TrkA receptors [35], insulin receptor IRβ subunits [59], TLR-4 [34, 61, 62, 64], and TLR7 and −9 [65]. Co-immunoprecipitation experiments using cell lysates from RAW-blue macrophage cells demonstrated that the 80 kDa NMBR isoform forms a complex with the active 88 kDa MMP9 isoform from naïve or lipopolysaccharide (LPS)-stimulated cells [62]. These data further validated that NMBR forms a complex with MMP9 on the cell surface of naïve cells. The report also showed that GPCR agonists (including bombesin, lysophosphatidic acid (LPA), cholesterol, angiotensin-1 and −2, and bradykinin) binding to their respective GPCRs induce Neu1 activity within 1 minute [62]. This prompt Neu1 activity was blocked by Gα_i_-sensitive pertussis toxin, the neuraminidase inhibitor oseltamivir phosphate, broad-range MMP inhibitors galardin and piperazine, anti-Neu1 and anti-MMP9 antibodies, and siRNA knockdown of MMP9. The rapid GPCR agonist-induced Neu1 activity verifies that sialylated receptors, including RTKs and TLRs, form a functional GPCR-signaling complex as depicted in Figure 1.

It has also been shown that insulin can mediate increases in MMP9 via insulin receptor (IR) activation [148], which fits well within this molecular signaling platform for insulin-induced receptors [59]. The study has also shown that insulin can induce MMP9 upregulation via the mitogenic signaling pathways, and renders the PI3K-dependent signaling pathway unnecessary. Indeed, the PI3K-dependent pathway is typically altered and is not required in insulin resistance [148]. The connection between GPCR and IR has also been demonstrated for β-adrenergic receptors tethered to IR in adipocytes [149–152]. These reports showed that insulin-bound IR stimulates the Tyr-350 phosphorylation of the β-adrenergic receptor, and that this process facilitates IR tethering to β-adrenergic receptor via growth factor receptor-bound protein-2 (Grb-2). This molecular signaling platform integrating the IR/β-adrenergic receptor/Grb-2 tripartite complex is critical for insulin-dependent activation of p42/p44 MAPK. These RTK–GPCR signaling platforms are reviewed in detail by Pyne and Pyne [153] and Abdulkhalek et al. [31], and thus, may play an essential role in tumorigenesis. Indeed, membrane lipid rafts are highly ordered membrane domains that are enriched in cholesterol, sphingolipids and gangliosides. They behave as major modulators of membrane geometry, lateral movement of molecules, traffic and signal transduction. Lipid rafts have recently been reviewed in detail as major platforms for signaling regulation in cancer [13].

## ROLE OF NEU1 SIALIDASE IN CANCER CELL SURVIVAL AND ACQUIRED CHEMORESISTANCE

The mechanism(s) by which cancer cells acquire resistance to chemotherapy is not well understood. An insight into the chemoresistance of PANC-1, Mia-PaCa-2 and ASPC-1 pancreatic cancer cells came from a study describing the aggressive and highly metastatic behaviour of pancreatic cancer due to an aberrant expression of nerve growth factor (NGF) and its high-affinity receptor, proto-oncogene TrkA [154]. We have reported that a Neu1-MMP9 crosstalk in alliance with neuromedin GPCR Gα_i_-signaling also regulates NGF induced TrkA activation, downstream cellular signaling, and cellular function [35].

Chemoresistance along with high rates of metastasis has been shown to contribute to the low survival rates of patients diagnosed with pancreatic cancer [155]. Currently, the standard of care for patients with pancreatic cancer is a chemotherapeutic agent called gemcitabine. Although treatment with gemcitabine shows higher success rates than any other chemotherapeutic used, such as 5-fluorouracil (5-FU) and cisplatin, patients receiving gemcitabine treatments only achieve a progression-free survival ranging from 0.9-4.2 months [156]. Given the poor response rate to gemcitabine, it has been suggested that pancreatic cancer cells develop rapid resistance to this drug [156, 157].

Within the last decade, studies have reported that there is a unique connection between drug resistance and epithelial-mesenchymal transition (EMT) [156, 158]. EMT in cancer cells is characterized by a loss of E-cadherin, cell-to-cell adhesion, and a promotion of cancer cell metastasis and progression. Creighton and colleagues have eloquently reviewed the role of EMT programming in cancer cell invasion and metastasis [159]. The report describes cells undergoing EMT that typically show both an increase in vimentin, N-cadherin, fibronectin, integrin αvβ6, and a decrease in E-cadherin, desmoplakin, cytokeratins, and occludin. In addition, several transcriptional suppressor families have been described that regulate EMT, including the zinc-finger proteins Snail1 and Snail2, the two-handed zinc-finger δEF1 family factors (δEF1/Zeb1 and SIP1/Zeb2), and the basic helix–loop–helix factors, Twist and E12/E47. Evidence also suggests that signals derived from the cellular microenvironment can regulate EMT, such as through cell–cell contacts mediated by families of transmembrane receptors and ligands expressed on adjacent cells. What is less clear is the relationship between EMT and chemoresistance in cancer. It is noteworthy that silencing of the two-handed zinc-finger δEF1 family factor, ZEB1, a transcriptional repressor of E-cadherin, actually restored the drug sensitivity in pancreatic cancer cells [160]. Other reports have indicated phenotypic changes which are consistent with EMT in breast, ovarian, and lung cancer cells that become resistant to drug therapy [156, 158, 161, 162]. Collectively, these results suggest a link between EMT and the acquisition of drug resistance, but the mechanisms behind this link may be complex and are not well understood.

There are reports that strongly implicate transforming growth factor-β (TGF-β) with EMT [163–167]. TGF-β is a multifunctional cytokine that is closely involved in regulating numerous physiological processes, but also functions as a powerful tumor suppressor in mammary epithelial cells (MECs), whose neoplastic development ultimately converts TGF-β into an oncogenic cytokine in aggressive late-stage mammary tumors [159]. Recent findings have implicated the process of EMT in mediating the functional conversion of TGF-β during breast cancer progression, suggesting that the chemotherapeutic targeting of EMT induced by TGF-β may offer new approaches in ameliorating metastatic disease [159, 168]. Katoh et al. [169] have reported that Hedgehog signaling activation indirectly leads to EMT through Notch, TGF-β signaling cascades, and a small non-coding RNA molecule, microRNA (miRNA), regulatory networks. For an example, Hedgehog signaling induces Notch ligand JAG2 upregulation for Notch-CSL-mediated SNAI1 upregulation, as well as TGF-β secretion for ZEB1 and ZEB2 upregulation via TGF-β receptor and NF-κB [169]. TGF-β-mediated down-regulation of miR-141, miR-200a, miR-200b, miR-200c, miR-205, and miR-429 results in upregulation of ZEB1 and ZEB2 proteins. Hedgehog signaling activation may indirectly lead to EMT through Notch, TGF-β signaling cascades, and miRNA regulatory networks [169]. Olive et al. have reported that inhibition of Hedgehog signaling enhanced the delivery of gemcitabine chemotherapy in a KPC rodent model of pancreatic ductal adenocarcinoma [170]. Collectively, it was proposed that if a drug could halt the process of EMT, it might also overcome chemoresistance, reduce metastasis, and thereby, improve the prognosis for patients diagnosed with pancreatic cancer.

When cancer cells utilize signaling initiated by EGFRs, they establish an anti-apoptotic state within the cell as well as to upregulate mitogenic, angiogenic and pro-invasive cellular mechanisms [171]. EGFR signaling has also been linked to EMT [172]. Therapeutic targeting EGFR and its inhibition can cause a reversal of EMT in human pancreatic cancer [172]. Other studies have suggested the potential role of growth factor receptor signaling in establishing chemoresistance of cancer cells [173–176]. Since EGFR signaling appears to be involved in both the acquisition of chemoresistance and the induction of EMT, it represents a prime therapeutic target.

We have reported that chronic treatment of human pancreatic PANC1 cancer cells with 0.01μM gemcitabine, 80μM cisplatin, or in their combination resulted in characteristic morphological changes with increased spindle shape morphology and cellular projections [46]. In addition, there were clear molecular alterations involving increased expressions of N- and VE-cadherin and a decreased expression of E-cadherin. These cadherin markers are consistent with other reports of EMT [177–179]. The report has also identified that oseltamivir phosphate has the ability to induce mesenchymal to epithelial transition (MET) both *in vitro* and *in vivo*. PANC1 chemoresistant cell lines (PANC1-GemR, PANC1-CisR, and PANC1-GemR/CisR) treated with oseltamivir phosphate resulted in the largest reduction of cell viability compared to PANC1 cells with the drug treatment alone. These results highlight the synergistic impact of oseltamivir phosphate and chemotherapeutics on chemoresistant PANC1 cell viability. This effect also suggests the capability of oseltamivir phosphate to reverse and increase the sensitivity of cancer cells to the chemotherapeutic agent to which they acquired resistance. Although oseltamivir phosphate has been shown to target and inhibit Neu1 sialidase activity associated with ligand-induced receptor activation on the cell surface [34], it may actually have broader specificities for other sialidases. With regard to chemoresistance of cancer cells, the therapeutic effects of oseltamivir phosphate could be due to a multitude of different molecular pathways. Connecting chemoresistance with Neu1 sialidase, another report found that MUC1 induces drug resistance in human (BxPC3 and Capan-1) and mouse (KCKO, KCM) pancreatic cancer cells [180]. These pancreatic cancer cells expressing high levels of MUC1 exhibited increased resistance to chemotherapeutic drugs such as gemcitabine and etoposide in comparison with cells that express low levels of MUC1. This chemoresistance was attributed to the enhanced expression of multidrug resistance (*MDR*) genes including *ABCC1, ABCC3, ABCC5* and *ABCB1* [180]. Of particular interest, the levels of the multidrug resistance-associated protein-1 (MRP1) encoded by the *ABCC1* gene were significantly higher in the MUC1-high cancer cells. In BxPC3 and Capan-1 cells, MUC1 upregulated MRP1 via an Akt-dependent signaling pathway, whereas in KCM cells, MUC1-mediated MRP1 upregulation was an Akt-independent mediated mechanism(s). It is unclear the reason(s) for this disparity in the cancer cells, but in KCM, BxPC3 and Capan-1 cells, the cytoplasmic tail motif of MUC1 associated directly with the promoter region of the *Abcc1/ABCC1* gene. This latter report provided evidence for a critical role of MUC1 directly regulating the expression of *MDR* genes in pancreatic cancer cells, and thus conferring drug resistance [180]. Since Neu1 sialidase activity was shown to regulate MUC1 [49], it would suggest that MDR might be one of mechanisms why PANC1-GemR, PANC1-CisR and PANC1-GemR/CisR cells are resistant. Oseltamivir phosphate targeting Neu1 may also impact on this MUC1-mediated MRP1 upregulated pathway in addition to its impact on EGFR [30] and other growth factor receptors. The therapeutic effects of oseltamivir phosphate could thus impact different molecular pathways as described above. Based on preclinical data using a mouse model of human pancreatic cancer, we proposed that Neu1 is a novel alternate anti-cancer target in restraining tumor neovascularization, growth, metastases, and macrophage-mediated tumorigenesis [30]. The premise is that Neu1 forms a complex with a broad range of glycosylated growth factor receptors including sensory TLR receptors [31].

## IMMUNE-MEDIATED TUMORIGENESIS AND THE ROLE OF MACROPHAGES

Several reports based on clinical studies have shown that patients with chronic inflammation may be at risk of developing cancer [181–186]. For example, patients with chronic pancreatitis or gastric carcinoma following infection with *Helicobacter pylori* may be predisposed at an increased risk of pancreatic cancer. Patients with Crohn's disease or ulcerative colitis may develop colorectal cancer [181, 183–186]. Research studies linking chronic inflammation with cancer have suggested that macrophage-mediated tumor initiation at these sites. Through their persistent inflammatory role during the process of chronic inflammation, macrophages can secret pro-inflammatory cytotoxic molecules, including reactive nitrogen (RNI) and oxygen (ROI) intermediates that result in tissue and DNA damage, development of mutations, and establish a defective p53 activity in the surrounding epithelial cells, and thus predisposing the microenvironment to oncogenic transformation and tumor onset [181, 182, 187]. Additionally, these tumor-associated macrophages (TAM) are noted to produce a series of cytokines including TNF-α, IL-1β, and IL-6 that predispose premalignant cells to survival signals, and thereby establishing macrophage-mediated tumorigenesis [183–186, 188].

In addition to their role in tumor initiation, macrophages have a unique ability to shift phenotypes during the course of tumor progression. As previously mentioned, macrophages appear to release factors that promote neoplastic transformation during tumor onset in a given tissue. Once tumors are established, macrophages will “switch” to an immunosuppressive phenotype that supports tumor vascularization, growth and metastasis [189, 190]. This shift in phenotype is characterized by the two distinct polarization states of macrophages: the classically activated M1, and the alternatively activated M2 macrophages [191]. M1 macrophages are pro-inflammatory cells characterized by the release of inflammatory cytokines and cytotoxic molecules. Generally, M1 macrophages play vital roles in clearing pathogens and in initial antitumoral responses. In contrast, M2 macrophages are immunosuppressive and have the ability to release high levels of anti-inflammatory cytokines, and support angiogenesis, tissue remodeling, and repair. M2 macrophages, as well as M2-like TAMs, are known to reside in established tumors and promote tumor growth and metastases. [192–194]. Thus, evidence suggests that the stage of tumor development in a given tissue determines the different display of macrophage subsets, with pro-inflammatory M1 macrophages in sites of tumor initiation, and tumor-promoting M2-like TAMs in established tumors. Evidence shows that once tumors develop, TAMs become immunosuppressive and have a defective ability (a) to release pro-inflammatory cytokines, (b) to present tumor-associated antigen, (c) to lyse tumor cells, and (d) to stimulate the antitumor functions of T cells and NK cells [192, 195, 196]. In some forms of human tumors, TAMs have also been reported to show defective release of IL-12 [197], and to increase secretion of anti-inflammatory cytokines such as IL-10 [197, 198], to influence tumor progression via the promotion of tumor vascularization, growth, survival, and metastasis [182, 192, 199, 200]. The role of TAMs in tumor neovascularization is thought to involve a wide array of pro-angiogenic factors and enzymes, including VEGF-A, VEGF-C, and MMP9 [182]. A review by Biswas et al. [201] highlights several molecular mechanisms of macrophages that are involved in tumor progression. In addition, the activation of NF-κB in macrophages can be mediated by cell surface and intracellular Toll-like receptors (TLR) [183–186, 188], the process of which has been shown to play major roles in cancer. We have reported for the first time that Neu1 sialidase clearly plays a central role in mediating cell surface and nucleic acid-induced intracellular TLR activation, and the interactions involving NMBR–MMP9–Neu1 cross-talk constitute a novel intracellular TLR signaling platform that is essential for NF-κB activation and pro-inflammatory responses [31, 61, 62, 65].

## NEU1 SIALIDASE REGULATES TUMOR NEOVASCULARIZATION, GROWTH, AND METASTASIS IN MOUSE MODELS OF HUMAN OVARIAN AND TRIPLE-NEGATIVE BREAST CANCERS

There is substantial evidence to indicate that the zinc-finger transcriptional factors Snail and Slug (the two-handed zinc factors ZEB1/dEF1 and ZEB2/SIP1 and the basic helix-loop-helix transcription factors Twist and E12/E47) play major roles in epithelial carcinoma plasticity [38–41], and tumor progression and invasiveness [42–45]. Since Snail is identified as a potent EMT mediator, others have reported that it controls the proteolytic activity of MMPs that contribute to the phenotypic changes associated with EMT and invasion [42]. Their data indicated that knockdown of Snail expression reduced the mRNA level of MMP-2 and suppressed the gelatinolytic activity of MMP-2 and MMP9 *in vitro*, and inhibited the catalytic activity of MMP-2 *in vivo*. It was proposed that Snail plays an essential role in upregulating the proteolytic activity of MMPs during invasion and metastasis. Others have provided additional confirmation for Snail in inducing MMP-1, −2, −7 and −14 in liver and squamous cell carcinoma lines [202] as well as MMP9 in Madin-Darby canine kidney (MDCK) epithelial cells [37]. Moreover, MMP9 has been shown to trigger the angiogenic switch during carcinogenesis [203]. However, the molecular mechanism(s) by which the Snail-MMP signaling axis functions in tumor neovascularization remained unknown until now.

As previously discussed, the signaling paradigm depicted in Figure 1 describes a GPCR-MMP9-Neu1 signaling axis that is induced by RTK ligand binding and receptor activation on the cell surface. The molecular interactions within this model are proposed to control key downstream tumor-specific mechanisms involved in cancer progression. The initial induction of GPCR and Ga_i_-signaling is an important process in this paradigm. Specifically, our data indicate that the neuromedin B GPCR might be involved in this process [30]. Indeed, Moody et al. [74] have also reported that the neuromedin B receptor regulates EGFR transactivation by a mechanism dependent on proto-oncogene tyrosine-protein kinase (Src) as well as MMP activation. We have also reported that different GPCR agonists can indirectly activate Neu1 through the intermediate MMP9 in order to induce transactivation of TLRs and subsequent cellular signaling [62]. Similarly, other reports have found a dramatic increase in the activity of MMP9 in gemcitabine-resistant pancreatic cancer cells [204], which fits well within our molecular signaling platform of Neu1-MMP9 cross-talk in regulating growth factor receptors.

The role of MMPs in cancer development, and specifically the role of MMP9, has been well documented [205]. Evidently, the function of transcriptional factor Snail controlling MMP9 expression may be critical in the initiation of his process. Indeed, our recent studies have shown that the induction of MMP9 by Snail occurs in ovarian A2780 cancer cells, and is able to regulate tumor neovascularization [206]. This Snail-MMP-9 signaling axis may be a regulator of the proposed signaling paradigm as previously described by us [31].

These signaling processes in tumors have also been observed in mouse models of human triple-negative breast cancer (TNBC). We have also reported that heterotopic xenografts of MDA-MB-231 tumors developed robust vascularization in RAG2xCγ double mutant mice [112]. Oseltamivir phosphate treatment at 50 mg/kg completely ablated tumor vascularization, tumor growth and spread to the lungs with significant long-term survival at day 180 post-implantation, exhibiting tumor shrinkage and no relapse after 56 days off drug. To date, there are no targeted therapies that are effective for TNBC, and the current state of treatment options is extensively reviewed by Rastelli et al. [207].

For A2780 ovarian [206] and TNBC MDA-MB-231 [112] cancer cells, we proposed that Snail may play an essential role in tumor neovascularization. Here, Neu1 might be an intermediate candidate connecting the Snail-MMP9 signaling axis in tumor neovascularization and in promoting the growth and invasiveness of human triple negative breast and ovarian cancers. Indeed, Bergers et al. [203] have reported that MMP9 triggers the angiogenic switch during carcinogenesis. An angiogenic switch paradigm has been proposed for (a) a mouse model of breast cancer by macrophages [190], (b) a balance between two distinct TGF-β receptor signaling pathways [208], and (c) in macrophages involving synergy between TLR-2, −4, −7, and −9 and adenosine A(2A) receptors [209].

## CONCLUSIONS

The multistage developments of cancer, including tumor onset, proliferation, angiogenesis, immune evasion, and metastases, collectively call upon an alternate and broad-range approach to combating the disease. Here, we present a novel GPCR-MMP9-Neu1 signaling model that may play unprecedented roles in tumor progression and a novel role for therapeutic targeting of the multistage tumorigenesis. The preliminary involvement of Snail within this model may provide the molecular mechanism that controls this process, and its regulation in tumor development and vascularization. This review summarizes the recent studies that identify the tumor-specific role of the structures within our model, and emphasizes the importance of Neu1 as a new target in cancer treatment. Indeed, the desialylation activity of Neu1 has been shown to regulate cancer growth, and its selective inhibition has demonstrated significant therapeutic results in murine models of cancer. Neu1 inhibition by oseltamivir phosphate has been shown to specifically increase E-cadherin expression and to decrease N-cadherin expression in pancreatic cancer [30, 46], triple-negative breast cancer [112] and in ovarian tumor models [206]. This shift in E- and N-cadherin expression may obstruct the occurrence of EMT in drug-resistant phenotypes, prevent cancer cell metastases, and improve the drug sensitivity of chemoresistant cells. In summary, the reports in this review implicate Neu1 as a novel therapeutic target in cancer therapy, and as a promising intervention in multistage tumor development.
